# miRNA Combinatorics and its Role in Cell State Control—A Probabilistic Approach

**DOI:** 10.3389/fmolb.2021.772852

**Published:** 2021-12-21

**Authors:** Shelly Mahlab-Aviv, Nathan Linial, Michal Linial

**Affiliations:** ^1^ The Rachel and Selim Benin School of Computer Science and Engineering, The Hebrew University of Jerusalem, Jerusalem, Israel; ^2^ Department of Biological Chemistry, Institute of Life Sciences, The Hebrew University of Jerusalem, Jerusalem, Israel

**Keywords:** CLIP-Seq, miRNA-target prediction, TargetScan, ceRNA, Markov chain, cell line, cell simulation, miRNA binding site

## Abstract

A hallmark of cancer evolution is that the tumor may change its cell identity and improve its survival and fitness. Drastic change in microRNA (miRNA) composition and quantities accompany such dynamic processes. Cancer samples are composed of cells’ mixtures of varying stages of cancerous progress. Therefore, cell-specific molecular profiling represents cellular averaging. In this study, we consider the degree to which altering miRNAs composition shifts cell behavior. We used COMICS, an iterative framework that simulates the stochastic events of miRNA-mRNA pairing, using a probabilistic approach. COMICS simulates the likelihood that cells change their transcriptome following many iterations (100 k). Results of COMICS from the human cell line (HeLa) confirmed that most genes are resistant to miRNA regulation. However, COMICS results suggest that the composition of the abundant miRNAs dictates the nature of the cells (across three cell lines) regardless of its actual mRNA steady-state. *In silico* perturbations of cell lines (i.e., by overexpressing miRNAs) allowed to classify genes according to their sensitivity and resilience to any combination of miRNA perturbations. Our results expose an overlooked quantitative dimension for a set of genes and miRNA regulation in living cells. The immediate implication is that even relatively modest overexpression of specific miRNAs may shift cell identity and impact cancer evolution.

## 1 Introduction

Mature microRNAs (miRNAs) are non-coding RNA molecules that regulate genes through base complementarity with their cognate mRNAs, at the 3′-untranslated regions (3′-UTR) (Moore et al., 2015). Within cells, miRNAs act by destabilization of mRNAs and interfering with the translation machinery (Chekulaeva and Filipowicz, 2009; Eichhorn et al., 2014). It was established that alteration in the relative abundance of miRNAs may lead to transition between cell states and the establishment of cell identity (Peláez and Carthew, 2012).

The human catalog of miRNA includes about 2500 mature miRNAs derived from ∼1900 genes (Kozomara and Griffiths-Jones, 2013). However, in each human cell, only a few dozens of miRNAs are expressed in substantial amounts. The miRNA distribution has a long tail of lowly expressed miRNAs. A reduced set of miRNA families (∼250 representatives) combines miRNAs with a substantial overlap in binding properties. In reality, ∼60% of the human coding genes are postulated as targets for miRNA regulation (Ha and Kim, 2014; Jonas and Izaurralde, 2015). Many miRNAs carry the potential for targeting hundreds of transcripts (Rajewsky, 2006; Balaga et al., 2012). Looking from the transcripts’ angle, at the 3′-UTR of an mRNA, there are tens of predicted miRNA binding domains (MBS) (Landgraf et al., 2007). Experimental results using CLIP-based deep sequencing protocols provide quantitative amounts of miRNAs and mRNAs in living cells (Li et al., 2014). Unfortunately, these protocols suffer from poor consistency (Lu and Leslie, 2016).

The quantitative aspect of miRNAs within living cells is understudied. It includes the stoichiometry on miRNA and mRNAs and the combinatorics of MBS. For a given mRNA, the composition and relative positioning of MBS along the transcript dictate the potential of a fruitful interaction (Jens and Rajewsky, 2015), but not necessarily the contribution of any specific miRNA to the overall suppression of gene expression (Agarwal et al., 2015). From the perspective of the miRNA, a fundamental player in the regulation is AGO, the catalytic component of the RNA silencing complex (RISC) within cells, and its availability (Wen et al., 2011; Janas et al., 2012). This many-to-many relation of the miRNA-mRNA network calls for developing a probabilistic model that will capture the design principle of miRNA regulation within the context of any cell type.

In this study, we present a stochastic, probabilistic model that operates at the cellular level. Furthermore, we substantiate a quantitative view on miRNA regulation that assesses the impact of changes in the quantities and diversity of miRNAs along with changes in cell behaviors. Technically, we applied the iterative simulator (called COMICS) on a selected human cell lines while exhaustively testing the outcome of *in silico* miRNA overexpression manipulations. We confirm the robustness of cells to the combinatorial effects of miRNA manipulations while calculating the retention level of each mRNA at the end of a simulation run (100 k iterations). We identify genes that are sensitive to the rate of mRNA degradation (i.e., cell dynamics) and others that respond to the actual elevation in the amounts of expressed miRNAs. In this study, we expose overlooked properties of miRNA regulation that are highly relevant to the maintenance of cell identity and the progression of cancer.

## 2 Methods

### 2.1 Probabilistic Map for miRNA-mRNA Pairing

The probabilistic framework for the interaction between miRNAs and their matched mRNAs was defined according to TargetScan (Agarwal et al., 2015). Accordingly, a high probability of miRNA-mRNA interaction (values ranging from 0 to 1) complies with numerous features from the sequence, secondary structure, and evolutionary conservation. Altogether, a complete miRNA-mRNA interaction table includes 8.22 M pairs covering as well poorly conserved interactions. We used a compressed version of the interaction table that reports on evolutionarily conserved miRNA pairs. This table includes 1,183,166 pairs which cover 18,953 genes and 289 miRNA families. Interaction scores were mapped to binding probabilities according to TargetScan score: *p* = 1–2^score.

### 2.2 Normalizations of mRNA Expression and miRNA Families

For the mRNA expression profile, we extracted data from RNA-seq experiments of HeLa cells that reliably report on 16,355 mRNAs and 539 miRNAs (Mahlab-Aviv et al., 2019). All genes pass the minimum threshold of >=1 reads (for experimental details see (Mahlab-Aviv et al., 2019)). Based on accepted quantification, we define a cell to display a 2:1 ratio of miRNAs to miRNAs, with a predetermined amount of 50 k and 25 k miRNAs and mRNAs per cell, respectively. Applying a strict threshold of ≥1 molecule per cell resulted in 110 miRNAs and 3666 mRNAs. We limited the expression level to 5 mRNA molecules per cell from a total amount of 25 k molecule (e.g., 0.02%), to improve the robustness of the analysis. Following this threshold, 753 genes remained for further analyses.

### 2.3 Probabilistic miRNA-mRNA Simulator

The input to COMICS (Competition of miRNA Interactions in Cell Systems) includes a normalized number of molecules from the RNA-seq results, and the values reported for the miRNA-mRNA interaction probabilities (see above). In each run, a random miRNA is chosen from the predetermined available miRNAs distribution. Next, a target is randomly chosen according to available targets’ distribution. mRNA that is already bound by miRNA molecules can still be a putative target for another miRNA if the two MBS do not overlap on the same molecule. This is defined as a minimal legitimate distance (≥50 nucleotides apart) between two neighboring RISC. Upon a binding event, the free miRNA and mRNA distributions are updated, with bound mRNA molecules marked as occupied. An occupied molecule (i.e., at least one bound miRNA) is removed after 1 k iterations following a successful binding event (to mimic the destabilization, leading to transcript degradation). Following mRNA removal, the bound miRNAs return to the general pool of free miRNAs.

#### 2.3.1 Configuration of COMICS

COMICS simulator supports a broad set of configurable parameters (see Mahlab-Aviv et al., 2019) that provide a high level of flexibility: 1) the number of total miRNAs; 2) the number of mRNA molecules in the cell; 3) the number of iterations to complete the run; 4) the number of iteration interval between the miRNA-mRNA binding event and the mRNA removal; 5) random removal of unbound mRNAs according to a predetermined decay rate of the mRNAs; 6) addition of newly transcribed mRNAs along with the iterations interval; 6) incorporation of alternative miRNA-target mapping. It is also possible to activate COMICS with a random set of genes, or a pre-existing iteration as a starting point, before the simulation run. In this study, we used default parameters. For mimicking cell manipulation: 7) miRNAs or genes overexpressed according to a selected multiplication factor. Specifically, we tested 7 multiplication steps (from x1 to x1000). If miRNA was undetected in the naïve cell, an arbitrary starting amount of 0.02% (the equivalent of 10 miRNA molecules per cell) is artificially added to the naïve cell (marked as x1).

#### 2.3.2 Analytical Methods

Statistical values for correlations were determined using standard Python statistical package. For annotation enrichment statistics and visualization, we used Gene Ontology platform (Huang et al., 2007). Clustering was performed by k-means classification method. We used the unsupervised Elbow method to test consistency within clusters by the percentage of variance explained. (i.e., the ratio of the between-group variance to the total variance). A change in the slope indicates the preferred number of clusters in that dataset. Standard statistical tests were applied to provide *p*-value for protein set comparisons.

## 3 Results

### 3.1 Assessment of a Probabilistic Approach for Cell States

Our previous study modeled the outcome of the miRNA-mRNA network in living cells by simulating the stochastic nature of miRNA regulation (Mahlab-Aviv et al., 2019). We observed that the relative ratio of miRNA to mRNA dictates the kinetics and the steady-state of expressed genes as measured by tracing the mRNA decay where no new expressed mRNA is considered.

In this study, we extend the analysis by clustering genes by their distinct kinetics and by testing the sensitivity of gene classes to changes in miRNAs’ relative quantities. The nature of miRNA regulation in living cells is depicted by the absolute quantities, composition, and stoichiometry of miRNAs and mRNAs (Balaga et al., 2012). Systematic analysis of the miRNA-mRNA interaction network shows that miRNA regulation operates under tight stoichiometric constraints in living cells. Classifying genes into sets that are unified by a common property, reduces complexity, and provides new insights on genes that are unified by their sensitivity to miRNA regulation.

Experimental data from HeLa cells for miRNAs and mRNAs are extracted from repeated NGS experiments (Mahlab-Aviv et al., 2019). A total of 539 miRNA types and 16,236 expressed mRNAs were mapped according to a predetermined expression threshold. As the molecular interaction of miRNA and mRNA within a cell is a stochastic process, we developed COMICS as an iterative simulator that attempts to capture such interactions (Mahlab-Aviv et al., 2019).

### 3.2 COMICS Performance

**FIGURE 1:**
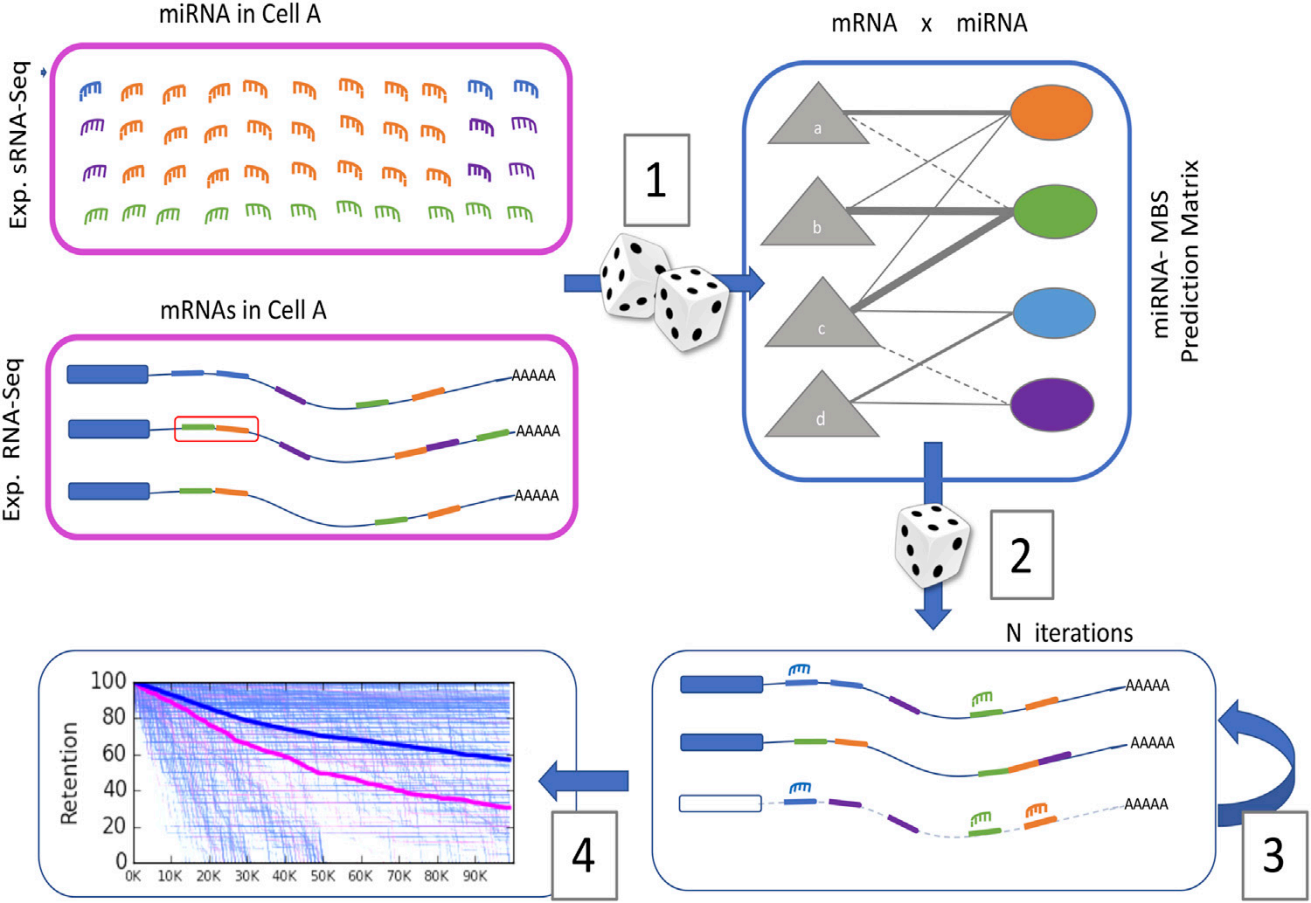
Schematic procedure of the probabilistic nature of COMICS (see text).

Figure 1 illustrates a scheme from a cellular perspective while focusing on the probabilistic framework. COMICS iterations capture the stochastic process in cells according to predetermined quantities and partition of miRNAs and mRNAs. The sampling process (Figure 1; Moore et al., 2015) is driven by the distribution of miRNAs and mRNAs that can be monitored experimentally (pink frames). Each mRNA is characterized by the types and positioning of its miRNA binding sites (MBS) on the transcript. The interaction table contains estimates of the probability-based scores for any pair of miRNA and MBS in the context of a specific mRNA. These calculated probabilities do not account for the fact that the expression of miRNAs and mRNAs are cell-type specific.

In each iteration, a miRNA is sampled randomly, according to the cell’s miRNA abundance and composition. Next, one of its target genes is chosen randomly according to the measured expressed mRNAs distribution. In the following stochastic step (Figure 1; Eichhorn et al., 2014), a randomly chosen miRNA and its target get paired by the sparse table of miRNA-MBS interactions (∼1.2 M pairs, see Section 2). Each miRNA-MBS interaction is associated with a probabilistic score that is a proxy for the level of confidence for that interaction and can be considered the probability of effective binding for any specific pair. Following a successful binding event, the distribution of the miRNAs and the mRNAs get updated (Figure 1; Chekulaeva and Filipowicz, 2009). Following a successful pairing, the status of the mRNA becomes “ready for degradation”. Upon binding, it may be engaged in the additional binding of miRNAs, but MBS interactions at close physical proximity are excluded. Our protocol supports cooperative binding on a target by imposing a degradation delay (e.g., 1 k iterations) that allows multiple miRNAs on the same mRNA. When an occupied mRNA is removed from the system, all its bounded miRNAs return to the miRNA pool. Consequently, the stoichiometry of miRNA to mRNA changes gradually with an increase in miRNAs to free mRNAs ratio. Note that additional variables that potentially impact miRNA regulation were omitted from the model for simplicity. These include cases in which binding of the miRNA to its target does not convert into mRNA degradation, instances of competitive endogenous RNA (ceRNA) (Thomson and Dinger, 2016), mRNA with an alternative or edited 3′-UTRs (Zhang et al., 2016), and subcellular partition of miRNAs (e.g., exosomes, nuclei, or cytoplasm) (Mahlab-Aviv et al., 2020).

The results of such a simulation are illustrated in Figure 1 (Peláez and Carthew, 2012). A decay rate for all genes is shown and the non-target (blue) and the genuine targets (pink) are signified with different dynamics and endpoints. Cell state is defined as the retention levels (%) of the unbounded genes at the end of the simulation run. The overall agreement of COMICS simulator protocol with experimental results was confirmed (Mahlab-Aviv et al., 2019).

The sensitivity of the values chosen to run COMICS was assessed by changing the total molecules in a cell, the initial ratio of miRNAs to mRNAs, the number of iterations required for reaching a steady-state, and more (see detailed in Mahlab-Aviv et al., 2019). Testing COMICS results by changing the input of miRNA-mRNA interaction matrix (see Figure 1) is fundamental for assessing the robustness of COMICS. To this end, we activated COMICS under two different mRNA-miRNA pair probabilities extracted from TargetScan and microRNA.org (Betel et al., 2010). The latter provides a MirSVR score for mRNA-miRNA pairs. The score is a result of a machine learning method that was trained on numerous contextual features allowing to rank MBS by their score for downregulation extracted from confirmed target sites.

**FIGURE 2:**
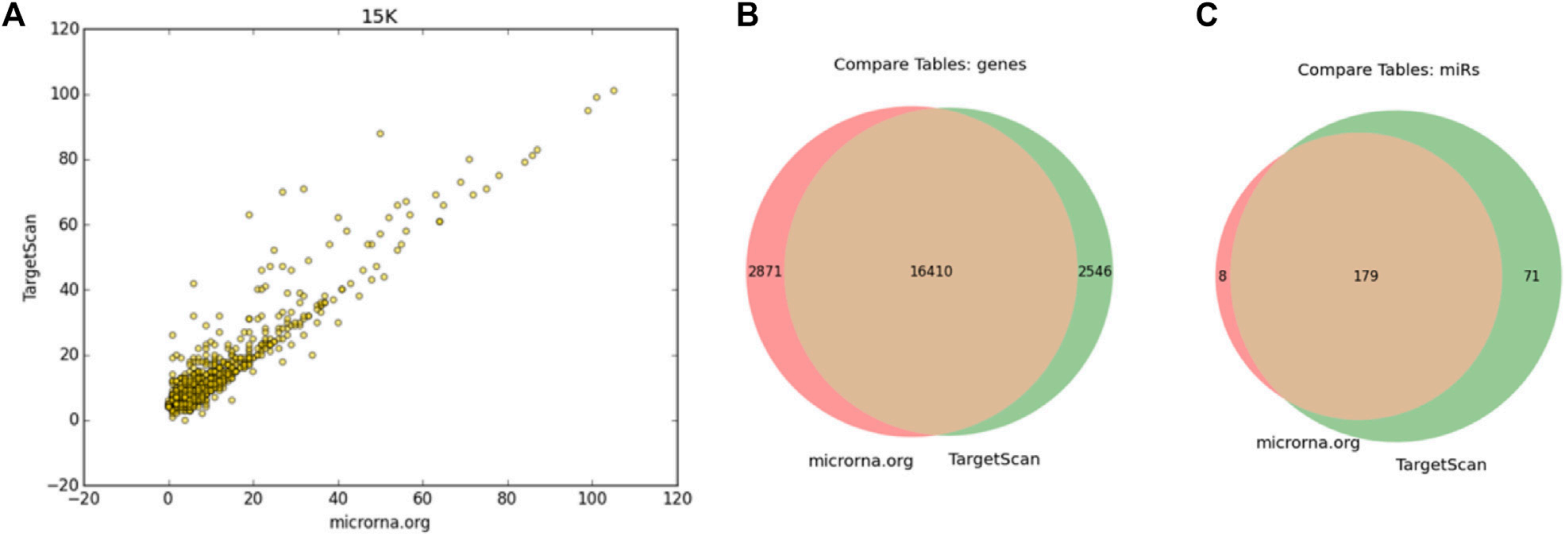
Comparing COMICS results by applying MBS-miRNA interaction table from TargetScan and microRNA.org **(A)** Correlation plot following 15 k iterations with r = 0.93. The interaction table from microRNA.org includes 1,097,065 pairs that covers 19,281 genes and 249 miRNAs, and TargetScan includes 1,183,166 pairs, 19,327 genes and 1741 miRNAs. Venn diagrams of the mRNAs **(B)** and miRNAs **(C)**.

Figure 2 shows the results of the comparison between the use of conservative TargetScan (see Section 2) and MirSVR scores following 15,000 COMICS iterations. A strong correlation (0.93) in using these alternative matrices confirmed the robustness of the analysis (Figure 2A). This is substantiated by the large overlap in COMICS results (at 100 k iterations) using these two alternative interaction matrices as input (Figures 2B,C).

TarBase v8 (Karagkouni et al., 2018) compiles the current knowledge on miRNA-target pairs from a broad range of experimental methodologies and conditions. This resource also includes cases on cell-type specific miRNA regulation. We performed a COMICS run (100 k iterations) and found a moderate correlation of the retention at the end of the run (382 genes shared genes; Pearson correlation = 0.38, *p*-value = 5.3e-14). Such correlation was completely lost following miRNA-mRNA pair randomization (Pearson correlation = 0.02).

We conclude that COMICS is quite robust to the use of a particular interaction miRNA-MBS scores (Figure 2) and corroborates with validated experimental knowledge on cell regulation by miRNAs.

### 3.3 miRNA Expression Dominates Cell Identity

Hundreds of cell lines were established for advancing cancer research (Ghandi et al., 2019). Each of these cell types is considered as a feature in the space of a multidimensional cell. In recent years, mRNAs and miRNAs expression profiles became available, thus providing a solid base to assess the contribution of cell-specific molecular landscape to tumorigenesis. The high correlation between biological samples of miRNA profiles across different cell types and platforms (e.g. (Lu et al., 2005)) was established. We utilize the molecular landscape of established cell lines (HeLa and HEK-293) that represent carcinoma lineages (of cervical and renal cell, respectively) to evaluate the dependency of miRNAs profile and COMICS outcomes.

**FIGURE 3:**
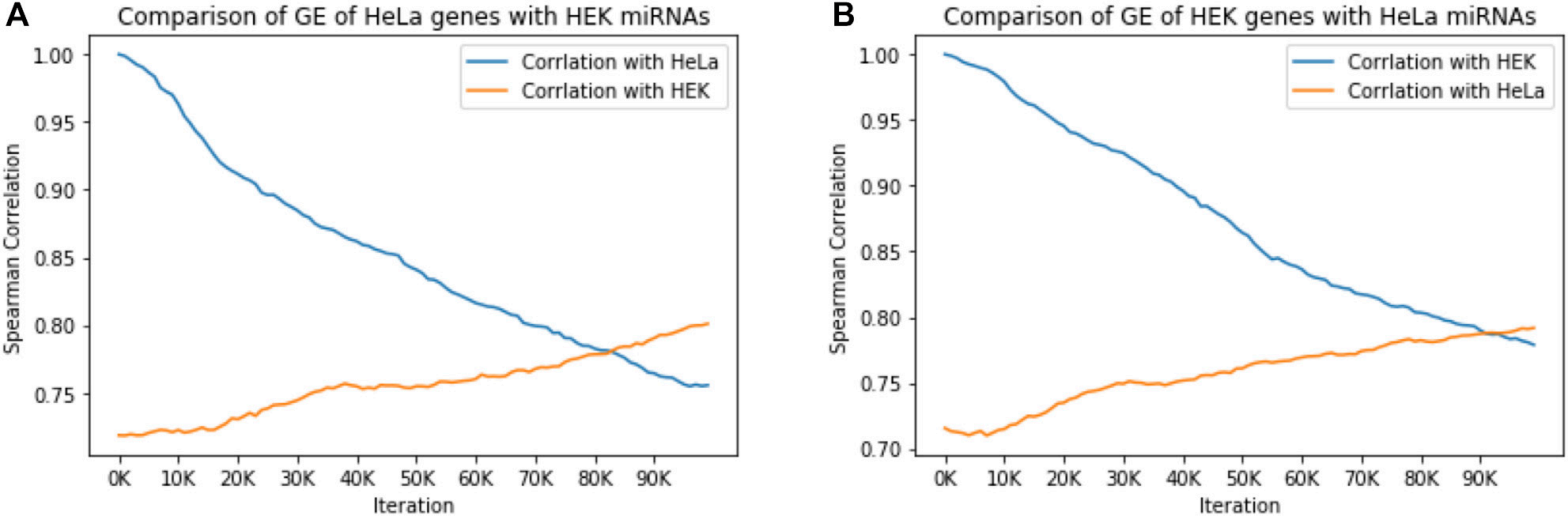
miRNAs govern cell identity. The effect of miRNA expression of one cell type on gene expression (GE) of a different cell type is measured by Spearman correlation of gene expression across the COMICS 100,000 iterations. **(A)** The correlation of HeLa regulated by its own miRNA expression profile (746 genes), with HeLa gene expression regulated by the miRNA expression profile from HEK-293 (514 genes). **(B)** The correlation of HEK-293 regulated by its own miRNA expression profile (516 genes), with HEK-293 gene expression regulated by the miRNA expression profile from Hela (514 genes). All correlation *p*-values are < 1e-15.

To this end, we normalized the absolute RNA-seq data to 50 k of miRNA and 25 k of mRNA per cell and monitored COMICS results along the run (100 k iterations). Assessment of the miRNA regulation in the cellular context relies on: 1) Expression of abundant miRNAs differs between cell lines (Mahlab-Aviv et al., 2019). 2) Only a handful of miRNAs account for 90% of the total cellular miRNA molecules in any specific cell. Figure 3 shows the results from comparing the trend of the gene expression by Pearson correlation across the 100 k iterations of a naïve cell and the cell in which the miRNA profile had been switched to that of the other cell type. Figure 3A shows the correlation between HeLa cells and an in-silico hybrid in which the miRNA profile was replaced with that of HEK-293 cells. Under such artificial setting, the correlation is markedly reduced (Figure 3A, blue). However, the reverse scenario in which the correlation between HeLa and HEK-293 was measures (Pearson correlation of 0.72, iteration = 0), we observed a monotonic increase in the correlation from 0.72 to 0.81 along the simulation run. Figure 3B shows a similar trend when the correlation of naïve HEK-293 is tested with respect to a hybrid setting (HEK-293 with miRNA profile of HeLa). The quantities of miRNAs in each of the two cell lines (normalized values) are listed in Supplementary Table S1. These results show that miRNAs largely dictate cell identity. We conclude that the molecular composition of miRNAs governs the dynamics and the steady state. Thus, tracing cell behavior across the progression of the simulation is informative.

### 3.4 Dynamic Gene Classes

**FIGURE 4:**
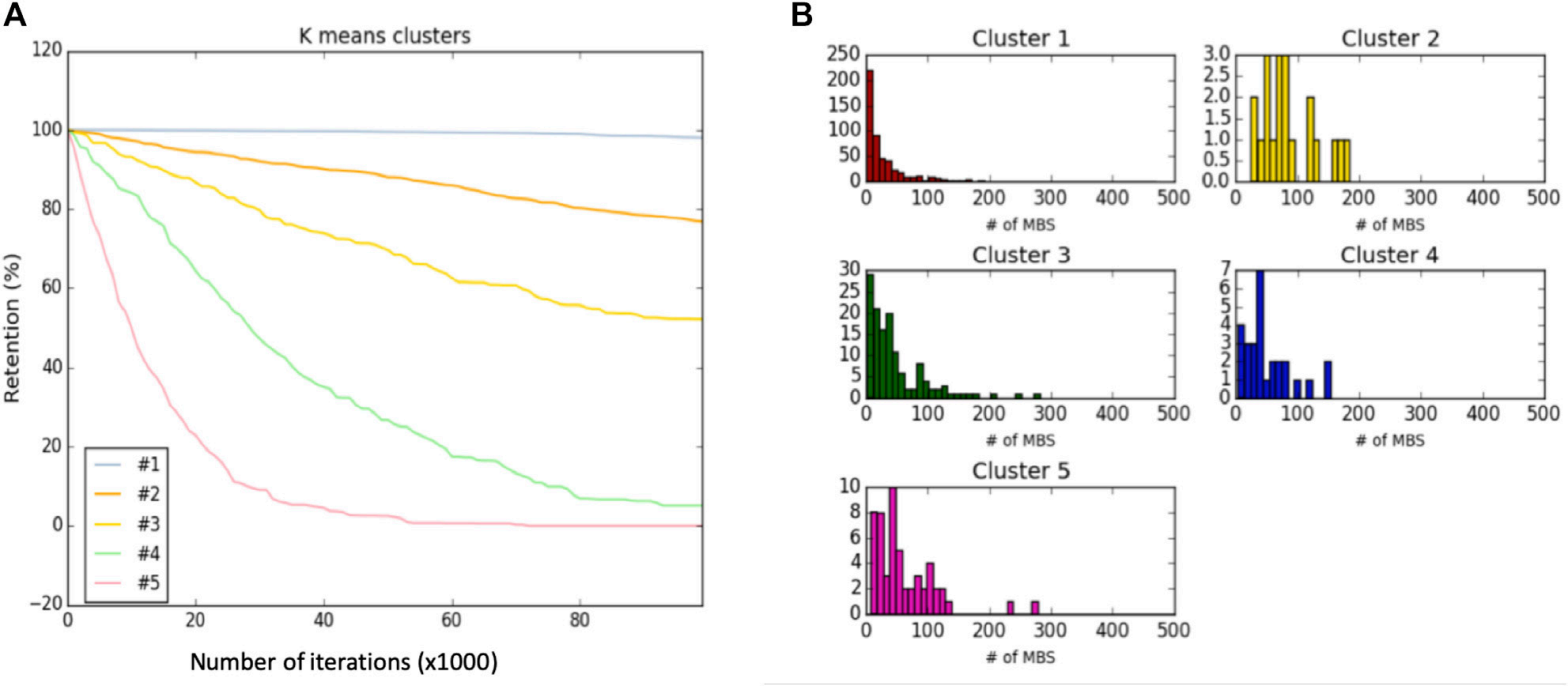
Dynamic classification and analysis. **(A)** Dynamic classes of Hela cells along 100 k iterations of a single simulation run. **(B)** The distribution of number of MBS per gene in the 5 dynamic classes (Dyn-class).

Figure 4 shows the results from HeLa cells and following k-means classification of genes according to their retention profiles throughout the simulation run. Figure 4A presents the clustering result for k = 5. We refer to each cluster as a Dyn-class. The average behavior of the dynamic classes of all genes is shown (total ∼750 genes). Genes are non-uniformly associated with the different dynamic classes, with cluster #1 covering 69% of the genes and only <4% being associated with the fast decaying cluster (cluster #5). Inspecting the decay rate of the lower retention clusters (cluster #4 and #5, 7% of total genes) show that despite differences in degradation rates, the endpoint almost coincides. While cluster #1, the most stable one, contains genes of the translation machinery, ribosomal subunits, chaperones, and cytoskeletal components, Cluster #5, with the lowest retention rate, contains genes that are enriched in the annotation of transcription regulators and splicing factors. We conclude that genes’ dynamics carries valuable functional information while reducing the complexity of the cell regulation model.

### 3.5 Exhaustive Perturbations of miRNAs

Accumulating evidence argues that an abrupt change in the expression of specific miRNA (or a set of miRNAs) may lead to a switch in cell identity tumorigenesis. Thus, we performed a set of manipulations in HeLa cells using a systematic approach covering all expressed miRNAs. We applied COMICS simulations by *in silico* overexpression of each of the 248 miRNA-MBS predictions (from TargetScan table, see Section 2). We multiply the basal abundance (x1) of each miRNA family by the following factors: x3, x9, x18, x90, x300, and x1000. For each such multiplication factor (f), final retention was computed, and cell state at the end of the COMICS run was monitored.

**FIGURE 5:**
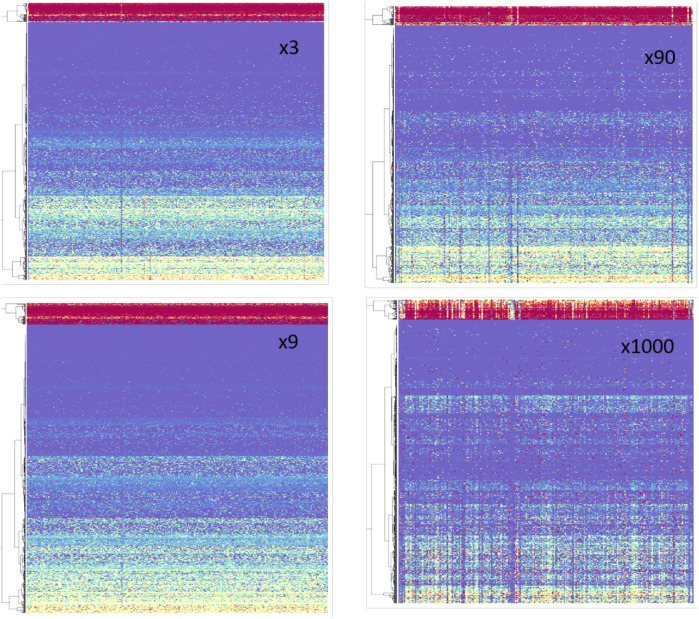
Retention values following *in silico* overexpression from HeLa cells. Rainbow color code: red = 0 to purple = 100% retention level at the end of the simulation run (100 k iterations). Each panel consists of 248 miRNAs (columns) and 750 genes (rows). The overexpression factors are indicated (x3 x9, x90, x1000). The miRNAs (columns) are sorted alphabetically, matrices are clustered by genes.

Figure 5 shows the pattern of the mRNA retention (%) for a matrix of miRNAs (columns) and genes. The panels show Mfij with factors x3, x9, x90, and x1000, where each of the listed miRNAs was overexpressed by the indicated multiplication factor. Therefore, each cell in the matrix Mfij is the final % of retention of gene i after 100 k iterations of COMICS for the overexpressed experiment of miRNA j (Figure 4). As the Mfij matrix reveals, genes are naturally clustered by their final retention level. For example, top “red” rows represent genes that were very sensitive to manipulation by any miRNA type (red = 0% retention; purple = 100% retention).

Several observations can be drawn from inspecting these matrices (Figure 5): 1) Large number of coordinated behaviors is evident. This is reflected by observing monochromatic rows across most miRNA columns. 2) As the overexpression factor increases (towards x1000), the pattern of the columns (i.e., specific miRNAs) becomes more informative and distinct. This is qualitatively seen as the increasing number of monochromatic columns at x90 and x1000 relative to their number in the moderate overexpression setting (x3 and x9). We present the result of the miRNA profile for overexpression of x300 (normalized) for 248 different miRNAs (Supplementary Table S2).

### 3.6 Perturbation by miRNA Overexpression of Pair Ratio Classes

Given the observation that genes behave similarly for their retention level at a broad range of Mfij, we tested the possibility of classifying genes by their sensitivity to miRNA abundance.

**FIGURE 6:**
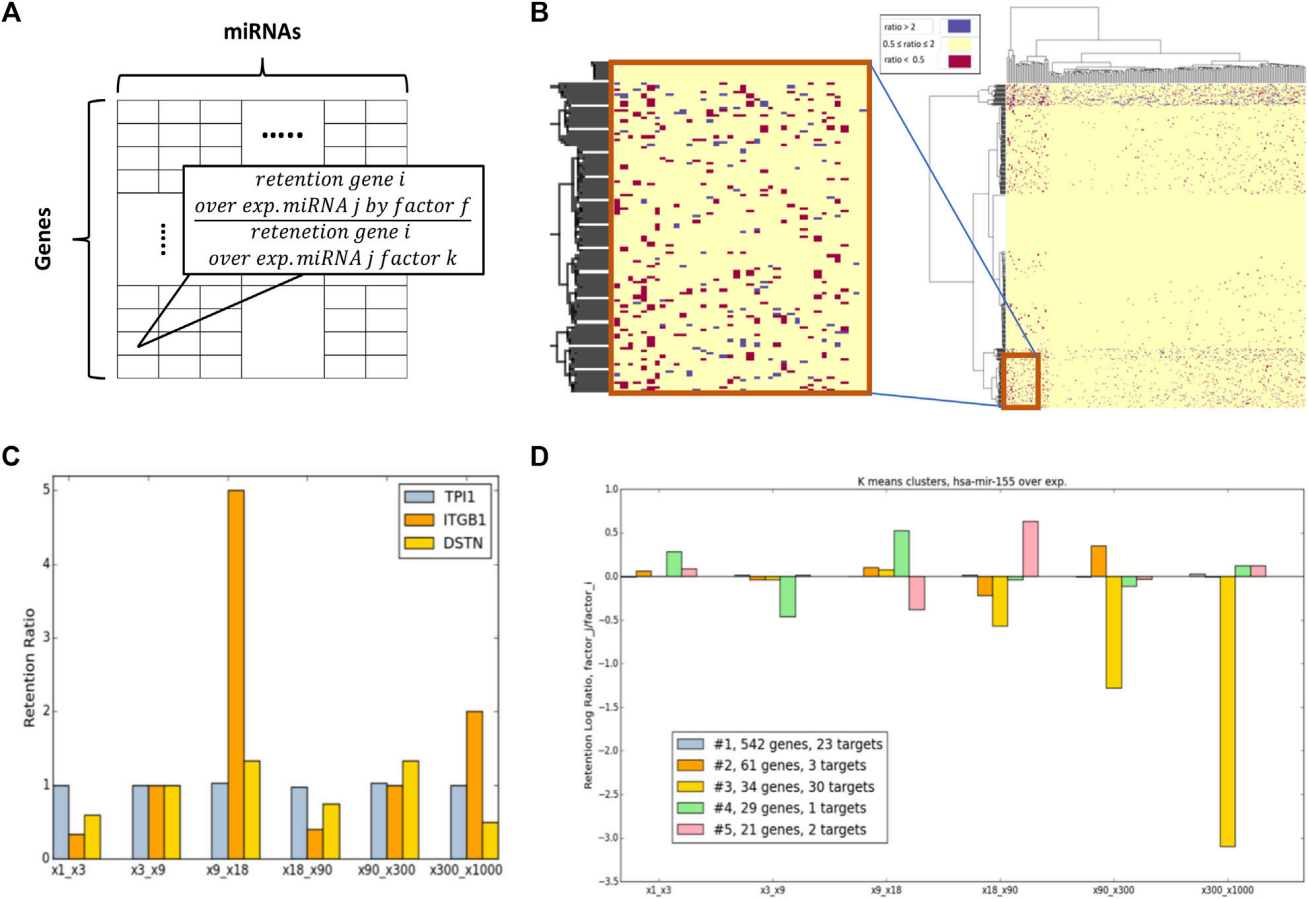
Overexpression ratio (OXR) classes. **(A)** Scheme for the presented Mfij/Mkij ratio retention matrix. **(B)** Ratio matrix for x90/x9 and a zoon-in for a small section. Colors only marked genes that show a significant ratio difference (by the predetermined differential folds of >2, and <0.5). **(C)** Examples of 3 selected genes according to the retention ratio from the 6-consecutive ratio-matrices. **(D)** A case study of hsa-mir-155 manipulation *via* OXR-clusters. A measure of the genes associated with five OXR-classes represented for 6-consecutive ratio matrices (x-axis). The numbers of expressed targets of hsa-mir-155 that are included in each OXR-class (including the numbers of direct targets) are indicated.

Following the relative changes of each gene retention by each miRNA, and an overexpression factor, we computed the retention ratio between any overexpression multiplications. Formally, we computed the value of Mfij/Mkij. This is the ratio of the retention of a specific gene (simulation at 100 k iterations) in a specific miRNA overexpressed by factor f, and its retention in the same miRNA overexpressed by factor k (Figure 6A). For visualization purposes, a discretization was applied for which ratio is > 2 folds. It implies that the retention of genes i in the overexpression of miRNA j by factor f is higher than its retention where miRNA j was overexpressed by factor k (Figure 6B, blue). However, a ratio that is < 0.5 implies that in factor f the gene is more prone to degradation for factor k (Figure 6B, red). Clusters with coherent behavior with respect to the ratio of two consecutive overexpression ratios were defined as OXR-classes.

Figure 6C illustrates the retention ratios of three selected genes (for illustrative purposes). It is shown that following overexpression of hsa-mir-155 the gene TPI1 (Triosephosphate Isomerase 1) remains stable throughout all tested retention ratios. As expected, TPI1 belongs to the dynamic class of genes that are extremely stable in the system (according to the Dyn-class). Different behavior is observed for ITGB1 (Integrin subunit beta 1) whose expression is very unstable and sensitive to a relatively minor change in amounts (the ratio of x18/x9). The non-monotonic behavior of ITGB1 and DSTN (Destrin, actin-depolymerizing factor) are evident.

Figure 6D illustrates the gene sensitivity as measured by the retention rate of different genes in the case of hsa-mir-155 for 6 pairs of factors: (x1, x3), (x3, x9), (x9, x18), (x18, x90), (x90, x300), and (x300, x1000). The results of COMICS retention for a specific miRNA overexpression were clustered by the K-means clustering algorithm (a cluster must contain >5 genes). The analysis reveals that OXR-classes display different sensitivity patterns for the pair-overexpression retention ratio. Figure 6D shows the partition of all genes into 5 clusters (marked #1 to #5). The majority of the genes (∼71%, cluster #1, colored grey) are indifferent to the levels of overexpression factors. However, the rest (∼29%) of the genes are sensitive to some extent to the overexpression factors that were used. For example, cluster #3 (Figure 6D, yellow) contains genes whose retention rate is drastically decreased as the overexpression factor increases (f). It is satisfying to note that most hsa-mir-155 expressed target genes (52%) belong to cluster #3.

**FIGURE 7:**
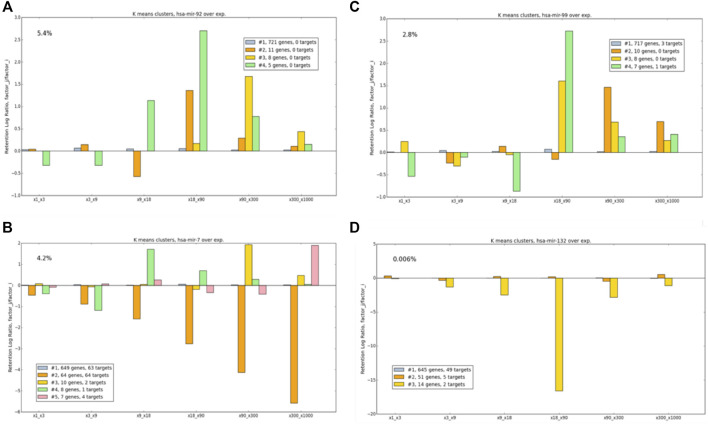
Example of OXR classes for different miRNAs, following manipulations of **(A)** has-mir-92, **(B)** hsa-mir-7, **(C)** hsa-mir-99, and **(D)** hsa-mir-132. The numbers of the targets of each tested miRNA are indicated (see color legend). miRNA expression (% of all miRNA in the cell) is marked. The values at y-axis are expressed by log2.

The illustration of Figure 6D was extended to show miRNAs that are candidates for strong cell-behavior dependency. Figure 7 shows 4 selected miRNAs according to their OXR-class across a 6-consecutive matrix ratio. The selected represented miRNAs are expressed at a different order of magnitudes. miRNAs that are highly expressed (e.g., hsa-mir-7, 4.2% of total miRNA in the cell), and others that are were analyzed and compared to low level expressing miRNA (hsa-mir-123, 0.006%).

Figure 7 exhibits several behaviors associated with the OXR-classes: 1) For all miRNAs, the largest OXR-class includes 82–91% of the analyzed genes. This cluster is quite stable, implying that most genes are insensitive to perturbation according to OXR. 2) The OXR-class that includes most targets of the subjected miRNA, a monotonic decrease is observed with a maximal effect seen for a ratio of the highest overexpression pair. This is shown for hsa-mir-7 in cluster #2. Note that for some miRNAs, no target is detected in the list of the analyzed genes (e.g., hsa-mir-92). 3) Some OXR-classes show a non-monotonic behavior that cannot be trivially anticipated, as shown for hsa-mir-99 cluster #4. 5) Some clusters show extreme increases or decreases in retention rates. The gene sets in such clusters exhibit high sensitivity to a specific miRNA abundance, as demonstrated for hsa-mir-132, cluster #3. Figure 7 illustrates OXR-classes for 6-consecutive ratio-matrices. A complete comparison and analyses are beyond this illustration. It includes each miRNA (total 248), for 21 pairs the 7 overexpression factors tested.

## 4 Discussion

The detailed quantitative considerations of miRNA and mRNA govern the dynamics and the steady-state of a gene expressed in cells (Bosson et al., 2014; Hausser and Zavolan, 2014). Cells’ behavior cannot be extracted from the direct measurements of miRNAs or mRNAs (Landgraf et al., 2007; Arvey et al., 2010). Still, insights on the regulation of gene expression by miRNAs in the complexity of the cells are improving as more experimental results become available (e.g., CLIP-Seq, CLASH (Li et al., 2014)) and the maturation of single-cell technologies. Most of the knowledge about specific miRNA in cancer samples relies on *in vitro* studies on the effect on an oncogene, tumor suppressor, or transcription factors. Despite progress in data collection that proposed a specific role of miRNAs in tumorigenesis, the underlying rules for regulation by miRNAs in the context of cell identity are still fragmented (Erhard et al., 2014).

The OXR-classes aim to capture the miRNA-dependent system dynamics (rather than the gene expression dynamics). We were able to cluster genes to their OXR-classes by performing hundreds of simulations that yield a robust assessment of cell states. For most instances, under all conditions, the majority of the expressed genes are not sensitive to the matrix-ratio measures. Namely, the final retention that is achieved in all conditions of overexpressed miRNAs is unchanged (Figure 7, y-axis = 0). In a smaller set of genes, a switch in the abundance of a specific miRNA may dramatically change target regulation (see examples in Figures 7B,D).

In this study, we consider two sets of gene classes: dynamic-classes (Dyn-class, Figure 4) and overexpression-ratio ratio (OXR-classes, Figures 6, 7). These two complementary types of classes capture different aspects of miRNA regulation. Results from the dynamic class show that genes which are likely to be successfully targeted are those with a relatively large number of MBS at their 3′-UTR (Figure 4B). However, Dyn-classes #2 to #5 are not distinguished by such features. Specifically, cluster #2, #3, #4, #5 are associated with 50.3, 71.6, 63.3, and 47.6 average MBS per gene.

There are numerous limitations in using COMICS to determine the sensitivity of cells to combinatorial regulation. For example, we assume all the measured miRNAs are accessible for regulation by miRNAs. However, a substantial fraction is not available in the cell cytosol. Partition of miRNAs in subcellular location is not addressed by our model (Mahlab-Aviv et al., 2020). Moreover, the TargetScan interaction table is restricted to major transcripts, and alternatively, spliced variants that potentially affect MBS are ignored. The addition of cell-specific genes list and gene versions will benefit the refinement of classification.

It was shown that miRNA profile is carefully regulated to promote and stabilize cell fate choices (Shenoy and Blelloch, 2014). Unfortunately, many experiments that use overexpression (and other perturbations like RNAi) do not measure or report the extent of miRNA overexpression. We have shown (Figure 5) that different genes exhibit different level of response to the absolute amount of the studied miRNA. We anticipate that inconsistencies among experimental results may be attributed to the missing overexpression factors.

Notwithstanding 2 decades of research in the miRNA field, basic principles remain unknown. Most current knowledge on the specificity of the miRNA-mRNA regulatory network is based on computational prediction tools (Peterson et al., 2014) that suffer from a flood of false positives (Pinzón et al., 2017). Experimental methodologies (e.g. CLASH and CLIP-Seq) that are based on capturing the interactions followed by sequencing show poor reproducibility (Lu and Leslie, 2016). miRNA regulation is often cell specific and changes in response to change in cellular conditions (as in differentiation, infection etc). Despite such variability, COMICS results agree with TarBase v8 (Spearman correlation = 0.38, *p*-value = 5.3e-14), arguing that the simplistic model implemented in COMICS provide valuable information that was further used to classify genes to various resultion classes. We suggest that a representation of genes by their Dyn- and OXR-classes can yield an accurate yet simple model for miRNA regulation. This study illustrates the use of COMICS results for gene classification. It further stresses the importance of a quantitative view for miRNA regulation modeling.

In pathological cells, such as in cancer, a quantitative change in the amounts of miRNAs is often the most significant molecular change observed in the early phase of cancer development (Peng and Croce, 2016). Assessing changes in the behavior of representative genes from OXR-classes could benefit cancer diagnosis. Some OXR-classes may serve as an indicator for a shift in cell states and identity. In all our classification approaches, only a very small number of coherent gene classes are reported. Cells likely display unexpected robustness concerning miRNA regulation (Ebert and Sharp, 2012). Revisiting our model on cell line encyclopedia will allow generalizing our observations to the collection of cancerous cells (primary and established) (Ghandi et al., 2019). The ability to classify genes according to dynamic overlooked features carries its potential to improve cell modeling and the understanding of cellular miRNA regulation in health and disease.

## Data Availability

The original contributions presented in the study are included in the article/Supplementary Material, further inquiries can be directed to the corresponding author.
