# Supplementary material for: miRNA Combinatorics and its Role in Cell State Control—A Probabilistic Approach
Source: Front Mol Biosci. 2021 Dec 21;8:772852. doi: 10.3389/fmolb.2021.772852 (PMC8724548; doi:10.3389/fmolb.2021.772852)
Supplement: Supplementary file 2 [file Table1.docx]

Supplementary Material

# Supplementary Tables

**Table S1:** Cell lines (HeLa and HEK-293) expression of miRNAs. Input for COMICS

**HeLa: miRNA Count (50k)** **HEK-293 Count (50k)**

mir-21-5p 16025 mir-7-5p 33268

let-7-5p 13582 mir-25-3p 2211

mir-92-3p 2717 mir-218-5p 1717

mir-30-5p 2336 mir-101-3p 1633

mir-7-5p 2110 mir-423-3p 1108

mir-100-5p 1719 mir-21-5p 1042

mir-27-3p 1654 mir-17-5p 1010

mir-99-5p 1416 mir-9-5p 880

mir-26-5p 747 mir-221-3p 826

mir-24-3p 667 mir-183-5p 632

mir-148-3p 566 mir-182-5p 509

mir-182-5p 489 mir-222-3p 492

mir-151-3p 457 mir-93-5p 489

mir-25-3p 436 mir-340-5p 461

mir-183-5p 365 mir-191-5p 454

mir-224-5p 347 mir-532-5p 418

mir-30-3p 314 mir-615-3p 295

mir-103-3p 309 mir-126-3p 224

mir-378-3p 299 mir-423-5p 213

mir-181-5p 285 mir-186-5p 187

mir-125-5p 285 mir-140-3p 171

mir-423-3p 282 mir-24-3p 162

mir-10-5p 279 mir-16-5p 145

mir-196-5p 182 mir-192-5p 143

mir-101-3p 180 mir-744-5p 134

mir-191-5p 166 mir-32-5p 110

mir-23-3p 149 mir-455-5p 96

mir-20-5p 131 mir-185-5p 81

mir-452-5p 85 mir-107 65

mir-320 74 mir-28-3p 65

mir-93-5p 73 mir-296-3p 56

mir-22-3p 73 mir-96-5p 55

mir-17-5p 72 mir-501-3p 46

mir-221-3p 67 mir-129-5p 31

mir-29-3p 65 mir-425-5p 28

mir-615-3p 58 mir-342-3p 26

mir-423-5p 57 mir-339-5p 26

mir-96-5p 45 mir-542-3p 26

mir-374-5p 44 mir-1271-5p 25

mir-186-5p 43 mir-421 25

mir-222-3p 39 mir-503-5p 24

mir-31-5p 39 mir-9-3p 24

mir-16-5p 37 mir-505-3p 23

mir-532-5p 36 mir-194-5p 20

let-7-3p 36 mir-375 20

mir-28-3p 34 mir-361-5p 19

mir-140-3p 34 mir-652-3p 17

mir-126-3p 31 mir-140-5p 16

mir-199-3p 30 mir-502-3p 15

mir-455-5p 26 mir-22-3p 14

mir-744-5p 25 mir-877-5p 12

mir-365-3p 19 mir-330-3p 11

mir-340-5p 18 mir-455-3p 10

mir-296-3p 15 mir-454-3p 10

mir-19-3p 14 mir-362-5p 8

mir-185-5p 14 mir-149-5p 8

mir-218-5p 13 mir-760 7

mir-425-5p 12 mir-330-5p 7

mir-130-5p 12 mir-1306-5p 7

mir-199-5p 11 mir-424-5p 7

mir-107 11 mir-31-5p 7

mir-143-3p 11 mir-132-3p 7

mir-32-5p 10 mir-195-3p 6

mir-192-5p 9 mir-126-5p 5

mir-15-5p 9 mir-532-3p 5

mir-151-5p 8 mir-28-5p 4

mir-22-5p 7 mir-708-5p 4

mir-149-5p 6 mir-17-3p 4

mir-106-5p 6 mir-187-3p 4

mir-139-5p 6 mir-143-3p 4

mir-129-5p 5 mir-331-3p 4

mir-200-3p 5 mir-486-5p 3

mir-361-5p 5 mir-212-5p 3

mir-146-5p 5 mir-188-5p 3

mir-450-5p 5 mir-296-5p 3

mir-140-5p 5 mir-346 2

mir-193-3p 4 mir-582-5p 2

mir-148-5p 4 mir-100-5p 2

mir-194-5p 4 mir-139-5p 2

mir-132-3p 3 mir-324-5p 1

mir-342-3p 3 mir-195-5p 1

mir-455-3p 3 mir-497-5p 1

mir-28-5p 3 mir-224-5p 1

mir-135-5p 3 mir-219-3p 1

mir-454-3p 3 mir-204-5p 1

mir-18-3p 3 mir-491-5p 1

mir-339-5p 3

mir-501-3p 3

mir-212-5p 3

mir-122-5p 3

mir-330-5p 3

mir-18-5p 3

mir-330-3p 3

mir-421 3

mir-505-3p 2

mir-582-5p 2

mir-195-3p 2

mir-34-5p 1

mir-219-3p 1

mir-296-5p 1

mir-381-3p 1

mir-486-5p 1

mir-877-5p 1

mir-193-5p 1

mir-138-5p 1

mir-335-5p 1

mir-9-5p 1

mir-502-3p 1

mir-503-5p 1

mir-652-3p 1
